# Supplementary material for: Cryo-EM Structure of Nucleotide-Bound Tel1ATM Unravels the Molecular Basis of Inhibition and Structural Rationale for Disease-Associated Mutations
Source: Structure. 2020 Jan 7;28(1):96–104.e3. doi: 10.1016/j.str.2019.10.012 (PMC6945111; doi:10.1016/j.str.2019.10.012)
Supplement: Document S1. Figures S1–S5 and Table S1 [file mmc1.pdf]

Structure, Volume *28*

Supplemental Information

Cryo-EM Structure of Nucleotide-Bound Tel1^{ATM} Unravels the Molecular Basis of Inhibition and Structural Rationale for Disease-Associated Mutations

Luke A. Yates, Rhys M. Williams, Sarem Hailemariam, Rafael Ayala, Peter Burgers, and Xiaodong Zhang

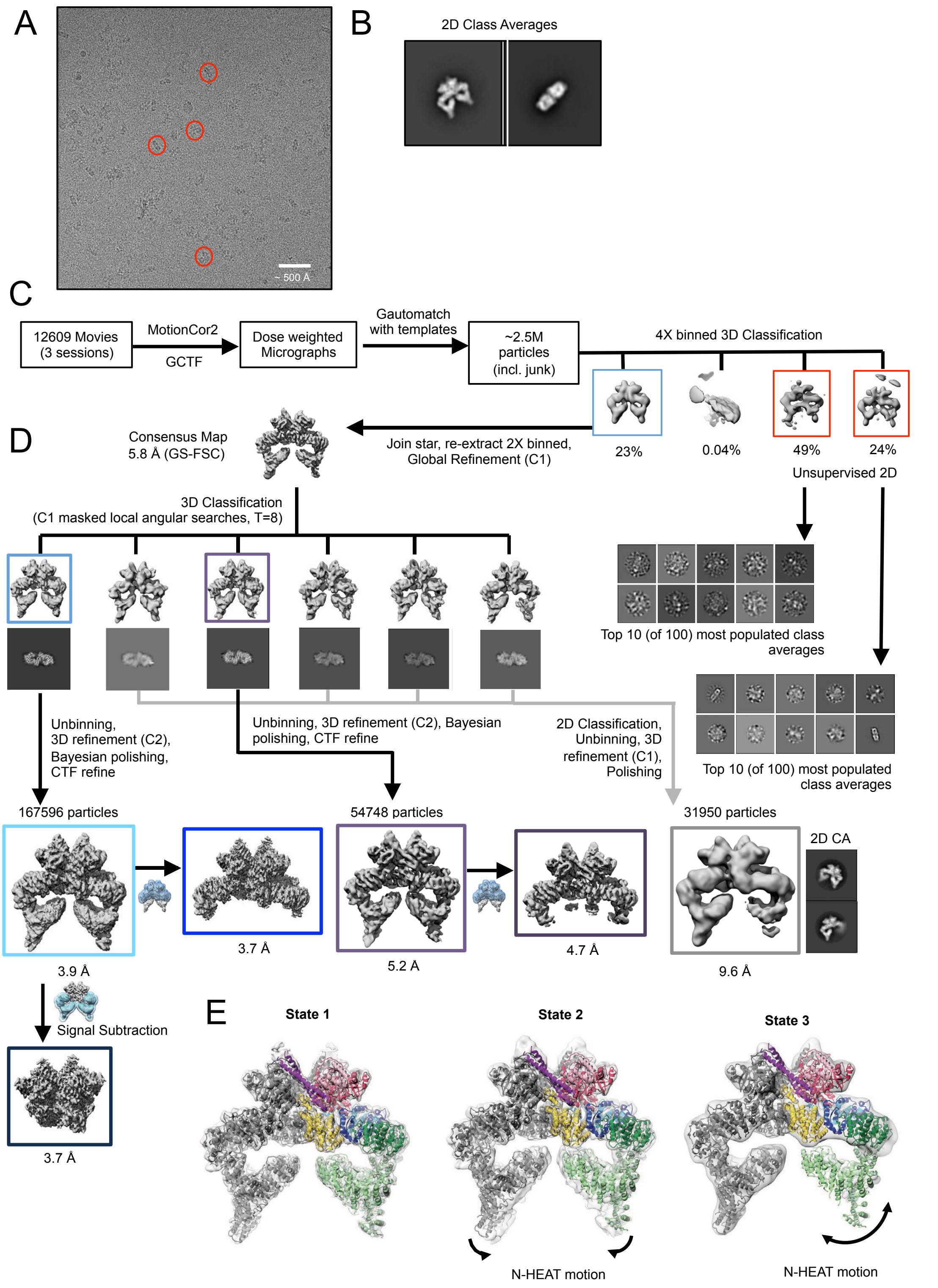

Fig. S1. (A) Representative micrograph, with a number of Tel1 particles circled showing distinct views of the protein in the raw image. (B) Major 2D class averages showing two predominant views. (C) Initial 3D classification to circumvent preferential orientation and processing of each class. (D) Processing arm of images corresponding to genuine Tel1 particles. Each refinement shown was performed by gold-standard methods in RELION-3.0. Slices of each 3D class are shown in this processing arm to illustrate classes with high-resolution features. (E) Tel1 dimer co-ordinates fitted into the three states obtained from the data processing tree. The FATKIN regions fit the density of each very well, however the N-HEAT regions display motion. **Related to Fig. 1.**

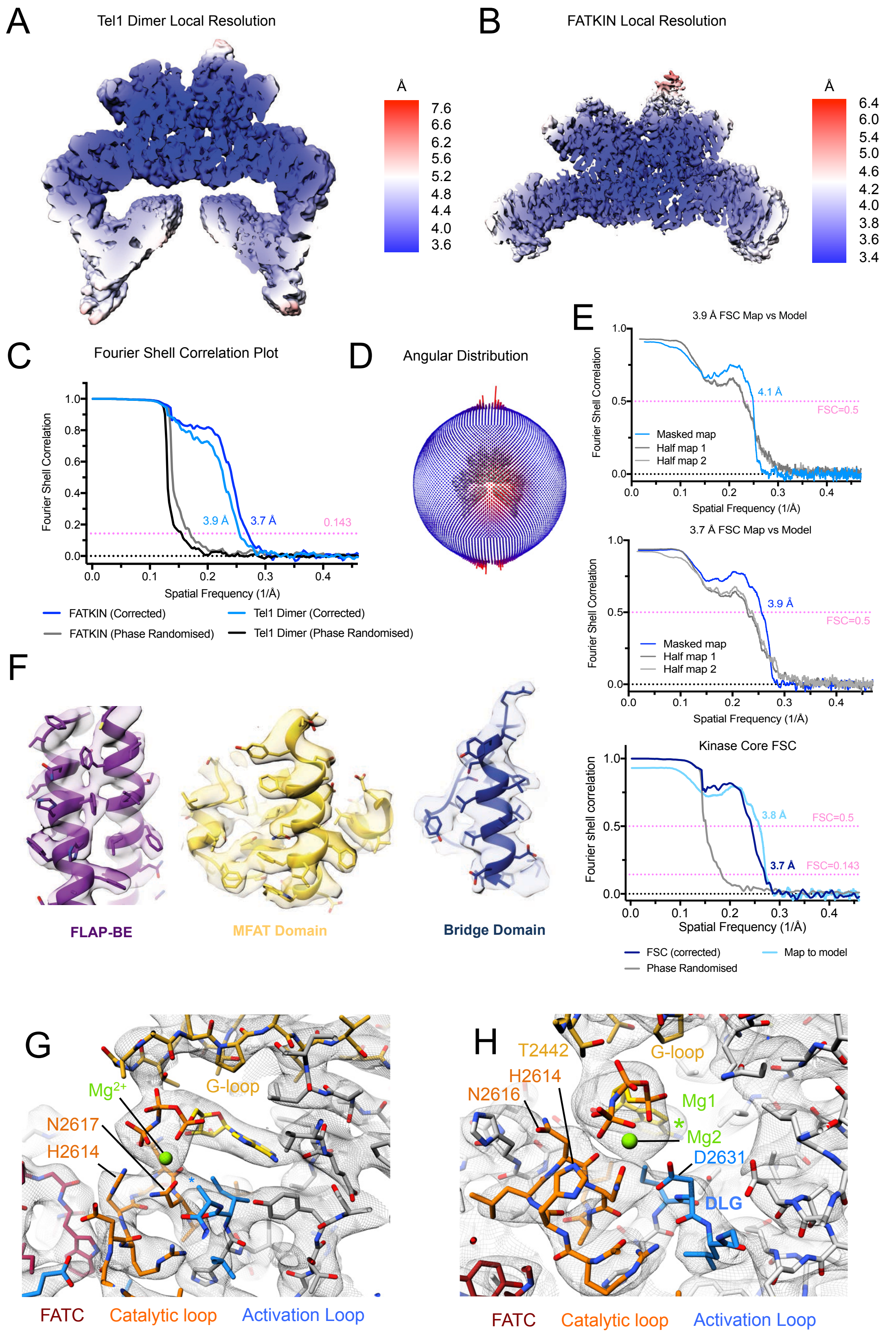

Fig. S2. Local resolution estimates of the (A) Tel1 dimer reconstruction and (B) FAT-KIN reconstruction calculated in RELION-3.0. A cut-away view into the core of the reconstruction is also shown. (C) Corrected Fourier Shell Correlation (FSC) curves. (D) Angular distribution of the Tel1 dimer reconstruction. (E) Map-to-model FSC curves for the reconstructions obtained. (F) Examples of density regions showing clear secondary structure and side chain details. (G-H) Close-up views of the density around the nucleotide and magnesium ion (Mg2). Green asterisk denotes position of Mg1. **Related to Fig. 1.**

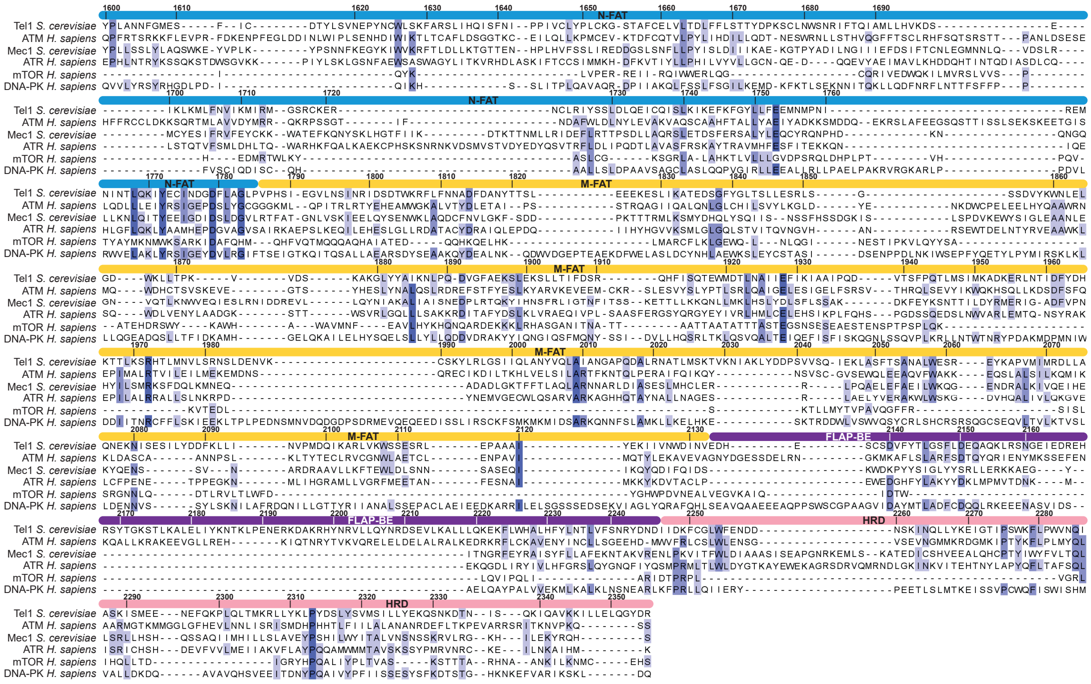

Fig. S3. Structure-based multiple sequence alignment of the FAT region of PIKKs. Tel1 residue numbers are shown above the sequence, with domain boundaries coloured as in Figure 1. **Related to Fig. 1 and 2.**

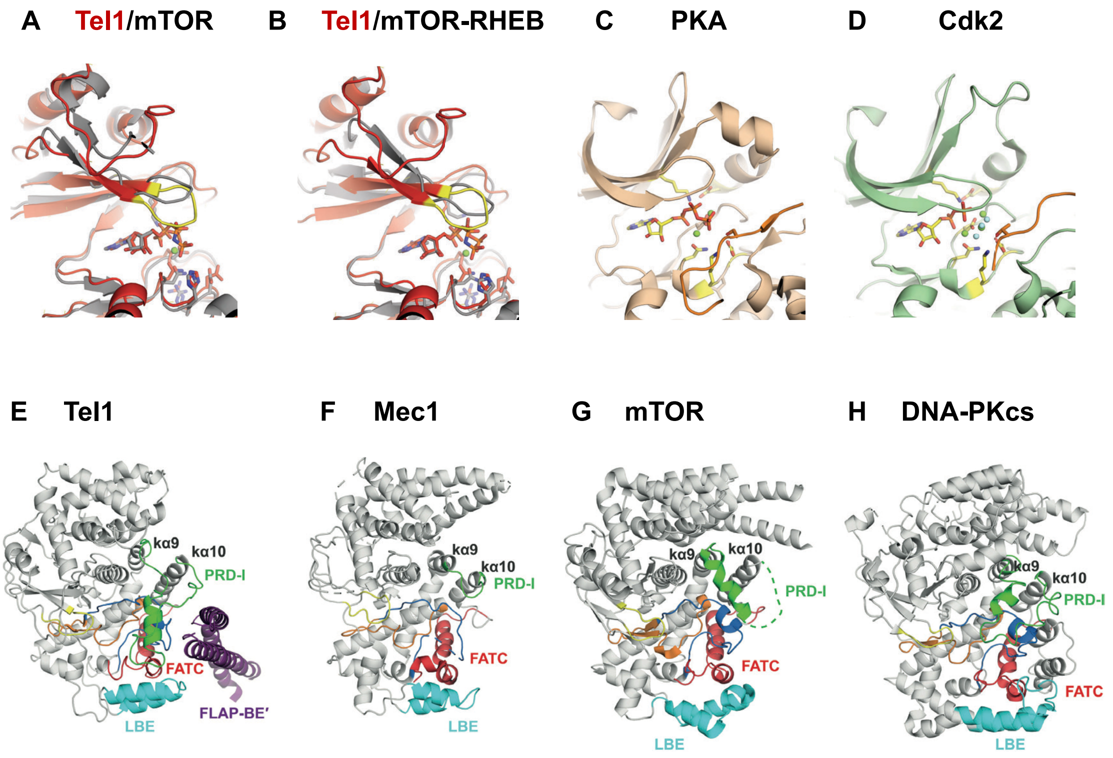

Fig. S4. Active site comparisons between (A) Tel1 (red) and mTOR (PDB-4JSV, grey); (B) Tel1 (red) and RHEB-activated mTOR (PDB 6BCU, grey). The active sites of two well-described kinase-peptide substrate complexes (C) PKA (PDB 3X2U) and (D) Cdk2 (PDB 3QHW) are shown for comparison. Structures of (E) Tel1, (F) Mec1, (G) mTOR, and (H) DNA-PKcs, showing structural divergence in the PRD-I, but conserved features in the catalytic sites, which are coloured as in Figure 3**. Related to Fig. 3.**

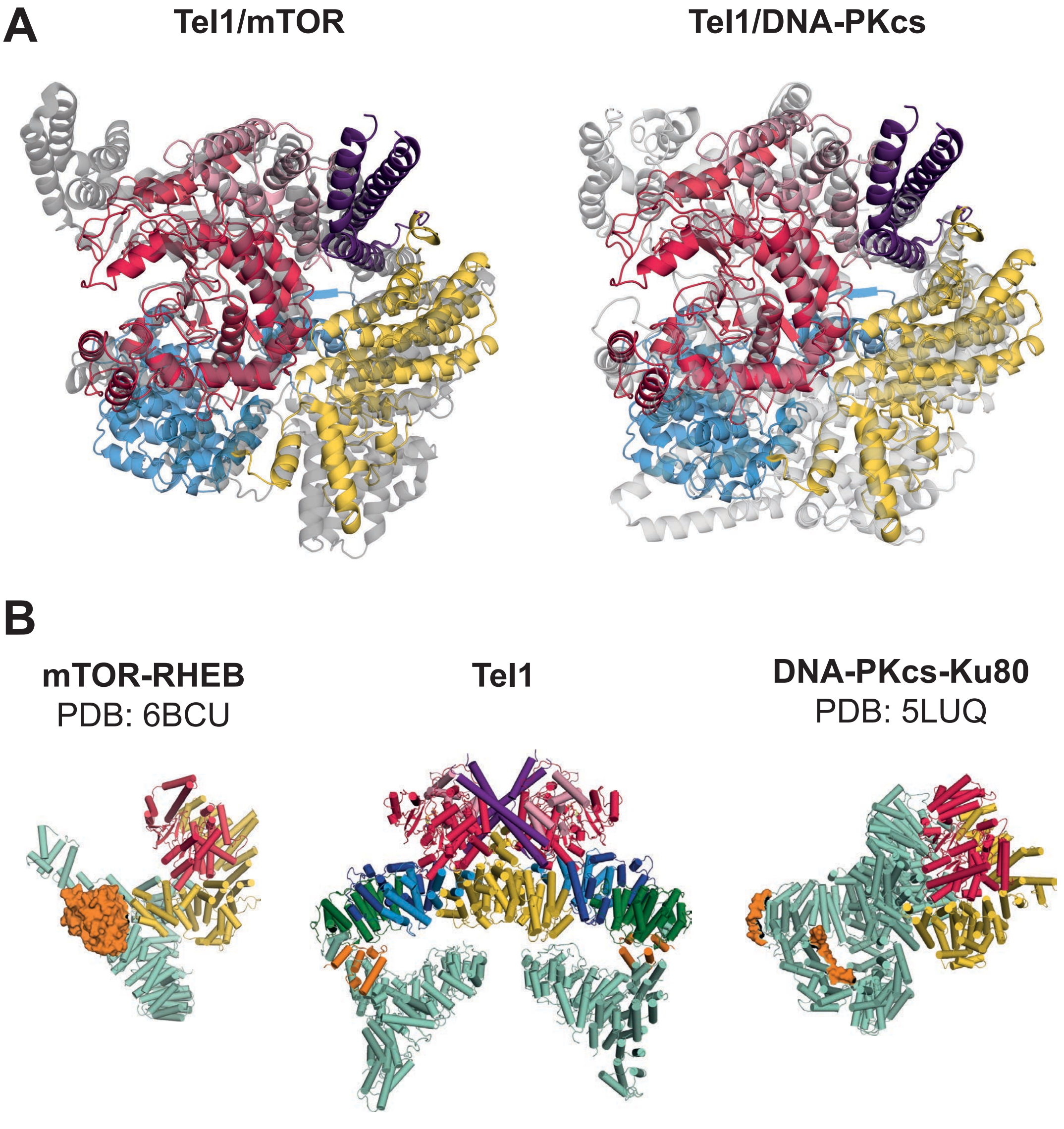

Fig. S5. Structural comparison of FAT-KIN regions between (A) Tel1 (coloured as in Figure 1) versus mTOR and Tel1 versus DNA-PK showing a conserved domain arrangement. (B) Activator-binding in mTORC1 (RHEB bind- ing) and DNA-PK (Ku70/80), where activators are rendered orange. Tel1 activator MRX binding site is unknown, but Xrs2 is shown to interact at the approximate region in Tel1, highlighted in orange. mTOR and DNA-PK are coloured by kinase (red), FAT (yellow) and HEAT repeats (green). All structures are aligned on the kinase domain. **Related to Fig. 3.**

Table S1. Tel1 hyperactivity mutations found in reference (Baldo et al., 2008), related to Figure 4.

**Variant**	**Mutation(s)**	**Region**	**Effect on Catalysis**	**Structural comments**
Tel1-hy385	N2692D	Kα9	Increase	
Tel1-hy394	I1765V	M-FAT	Increase	Could prevent allosteric activation
	K1879N	M-FAT		
	N2004S	M-FAT		
	R2675G	Kinase C-lobe		
Tel1-hy680	Q2764H	Kinase C-lobe	Increase	
Tel1-hy909	A2287V	HRD	Increase	ATM K3016 is Acetylated during activation
	I2336T	HRD		
	K2751R	Kinase		
Tel1-hy184	F1752I	M-FAT	None	PRD-I conformation may be altered
	D1985N	M-FAT		
	E2133K	M-FAT/FLAP-BE		
	R2735G	Close to PRD-I		
	E2737V	Close to PRD-I		
Tel1-hy628	N2185H	FLAP-BE loop (Not modelled)	None	*Cancer mutation, see below
	G2252C*	HRD packs against Kα9		
Tel1-hy544	F2576V	Kinase C-lobe	Reduction	
**ATM Disease-Associated Mutations**				
**ATM mutation**	**Tel1**	**Region**	**Disease**	**Structural comments**
G2867R	G2609	Kinase-Catalytic loop	AT	Disrupt activation and catalytic loop
R2849P	K2591	Kinase domain	AT	Kinase - M-FAT interface
F2827C	I2569	LBE (Kα4c)	AT	LBE contacts PRD
D2625E/A2626P	E2371/M2372	Kα1	AT	Disrupts N-lobe
V2424G	L2180	FLAP-BE	AT	Likely contacting PRD
Y2470D	Y2232	FLAP-BE	AT	Disrupts FLAP-BE
A2067D	K1833	M-FAT, Dimer interface	AT	
D2016G	D1780	M-FAT	AT	Interface with C-lobe
R2227C	R1973	M-FAT	AT	
V1913G	I1710	N-FAT	AT	N-FAT/M-FAT boundary
N1983S/Y	~E1763	HEAT repeats	Cancer	See R2832C
C2337R	I2102	Close to FLAP-BE	Cancer	M-FAT/FLAP-BE junction
E2423G	A2179	FLAP-BE	Cancer	Likely contacting PRD
R2443Q	R2209	FLAP-BE	Cancer	Holding FLAP-BE helices together
E2444K	Q2206	FLAP-BE	Cancer	
D2448Φ/N	D2210	FLAP-BE	Cancer	
R2453H/P	K2215	FLAP-BE	Cancer	

S2489F	G2252	HRD	Cancer	Packs against Kα9. Likely similar effect as mutation Y2954C in Kα9
G2694R/E	Gly-rich loop		Cancer	Disruption of active site
G2695X				
P2699X				
D2708N/Y	Hairpin at rear of Gly-rich loop			
G2709D				
D721X (+ neighbours)	Kα3 (PKA αC equivalent)			
N2875T/S	Catalytic loop (Mg2-binding)			
D2289X	DLG motif (Mg1-binding)			
L2890X				
G2891X				
R2832C	R2574	Kα6	Cancer	Likely interacts with region around P1759. ATM has an extended helix here, could be linked to allostery. This region also contains N1983S/Y
Y2954C	W2696	Kα9	Cancer	Beginning of PRD, packs against HRD. See S2489F
R3008H/C	R2743	Kα10	Cancer	Helix after PRD
